# Colorectal Cancer Risk Factors and Screening Among the Uninsured of Tampa Bay: A Free Clinic Study

**DOI:** 10.5888/pcd18.200496

**Published:** 2021-02-25

**Authors:** Ethan Y. Song, Justin Swanson, Artish Patel, Madeline MacDonald, Alexandra Aponte, Noura Ayoubi, Lucy Guerra, Eduardo Gonzalez, Rahul Mhaskar, Abu-Sayeef Mirza

**Affiliations:** 1 University of South Florida, Morsani College of Medicine, Tampa, Florida; 2 University of South Florida, College of Public Health, Tampa, Florida; 3 University of South Florida, Tampa, Florida; 4 USF Morsani College of Medicine, Department of Internal Medicine, Tampa, Florida; 5 USF Morsani College of Medicine, Department of Family Medicine, Tampa, Florida

## Abstract

**Introduction:**

Uninsured patients with low socioeconomic status are at high risk for developing colorectal cancer (CRC), and data on risk factors and prevalence of CRC in this population are limited. The purpose of this study was to assess the risk factors for CRC in uninsured patients from free clinics in the Tampa Bay area of Florida.

**Methods:**

We conducted a retrospective cohort study among patients 50 years or older who were provided service at 9 free clinics in the Tampa Bay area between 2016 and 2018. Demographics, chronic disease characteristics, and screening data were collected via a query of paper and electronic medical records.

**Results:**

Of the 13,982 patients seen, 5,139 (36.8%) were aged 50 years or older. Most were female (56.8%), non-Hispanic White (41.1%), and unemployed (54.9%). Patients with CRC screening were more likely to be employed compared with patients without CRC screening (54.4% vs 44.4%, *P* = .01). Within the cohort, 725 (22.7%) patients were active smokers, 771 (29.2%) patients currently consumed alcohol, and 23 patients (0.4%) had a history of inflammatory bowel disease. Patients had a median body mass index of 29.4 (interquartile range, 25.4–34.2) kg/m^2^, and 1,455 (28.3%) had diabetes. Documented CRC screening was found among 341 (6.6%) patients.

**Conclusion:**

Uninsured patients had a high prevalence of CRC risk factors but a low reported screening rate for CRC. Free clinics are uniquely positioned to provide patients at high risk for CRC with strategies to decrease their risk and to be screened for CRC.

**Summary**

**What is already known on this topic?**

Patients with low socioeconomic status have a high likelihood of developing colorectal cancer (CRC) due to associated risk factors and lower reported rates of screening.

**What is added by this report?**

This study is among the first to analyze the prevalence of CRC risk factors and screening rates of low-income and uninsured patients at free clinics in Florida.

**What are the implications for public health practice?**

Community-based health centers and free medical clinics are uniquely positioned to treat and care for this vulnerable population through the development of sustainable and cost-effective primary and secondary prevention strategies.

## Introduction

Colorectal cancer (CRC) is the third leading cause of cancer-related deaths in the United States (1) and is most prevalent in patients aged 50 years or older. Despite significant strides in overall cancer survival, several factors such as low income, lack of insurance, and being in a racial or ethnic minority group prevent many Americans from receiving optimal care (1–3).

Patients with low socioeconomic status have a higher likelihood of developing CRC because of associated risk factors such as alcohol intake, obesity, and smoking (4). Another social determinant of health associated with poor outcomes in patients is lack of health insurance (2). It is well documented that cancer screening rates are lowest in people without health insurance, which leads to high numbers of late-stage cancers (5–7). Patients with Medicaid or those who are uninsured are more likely to have metastatic disease as well as lower rates of definitive surgery and resection (8). Furthermore, patients of racial and ethnic minority groups experience higher incidence and mortality rates of CRC compared with White patients (3,6).

Community-based health centers (CHC) and free medical clinics (FMC) provide primary care services to a large proportion of underinsured and uninsured individuals. They serve as first-line care for the prevention and management of many CRC risk factors such as diet, smoking, alcohol use, and type 2 diabetes (9). However, data on the burden of CRC risk factors in patients of these clinics are limited. The purpose of this study was to assess the prevalence of known risk factors and screening rates of CRC in low-income and uninsured patients of free clinics around Tampa Bay, Florida.

## Methods

We included all uninsured patients served at 9 free clinics in the Tampa Bay area of Florida from January 1, 2016, through December 31, 2018, in this retrospective cohort study. We included patients aged 50 years or older served at any point during the study period, on the basis of US Preventive Services Task Force guidelines that recommend screening for CRC starting at age 50 because of the increased risk of colorectal cancer in this age group (10). We obtained data from paper and electronic medical records and used REDCap software for analyses (11). We compared patients who had documented CRC screening and those who did not by socioeconomic variables (ie, age, sex, race/ethnicity, employment status) and known CRC risk factors, including biometrics (ie, weight and body mass index), alcohol or tobacco use, and comorbidities (ie, diabetes and inflammatory bowel disease [IBD]). We present numeric variables as median (interquartile range [IQR]) and categorical variables as number (%). We used Mann–Whitney-Wilcoxon tests for numeric variables and χ^2^ tests for categorical variables; missing values were not included in tests of significance. Significance was set at *P* < .05.

All participating clinics consented to the use of their data. This study was approved by the University of South Florida institutional review board.

## Results

Of the 13,982 patients seen during the study period, 5,139 (36.8%) were aged 50 years or older and included for further analysis (Table 1). Of those with nonmissing demographic data, most were female (n = 2,896, 56.8%) and unemployed (n = 1,327, 54.9%), and nearly equal proportions were non-Hispanic White (n = 1,649, 41.1%) and Hispanic of any race (n = 1,639, 40.8%). Of those who reported their smoking status, 725 (22.7%) were active smokers, and 594 (18.6%) were past smokers (Table 2). Current and past smokers reported a median history of 15 pack-years (IQR, 5–35 pack-years). Of those who reported their history of alcohol consumption, 771 (29.2%) were active consumers, and 192 (7.3%) were past consumers. The median body mass index (BMI, kg/m^2^) of patients was 29.4 (IQR, 25.4–34.2). The sample included 1,455 (28.3%) patients with diabetes and 23 (0.4%) patients with IBD.

**Table 1:** Demographics of Uninsured Patients Serviced at 9 Free Clinics in Tampa Bay, Florida, 2016–2018^a^

Demographic Characteristic	All Patients (N = 5,139)	No CRC Screening (n = 4,798)	CRC Screening (n = 341)	*P* Value
**Age, median, y (IQR)**	58 (54–62)	58 (54–62)	58 (54–61)	.35
**Sex**				
Male	2,206 (43.2)	2,074 (43.6)	132 (38.7)	.09
Female	2,896 (56.8)	2,687 (56.4)	209 (61.3)	
Missing	37 (0)	37 (0)	0	
**Race/ethnicity**				
White	1,649 (41.1)	1,504 (40.6)	145 (47.1)	.04
Black	507 (12.6)	465 (12.5)	42 (13.6)	
Asian	194 (4.8)	183 (4.9)	11 (3.6)	
Hispanic, all races	1,639 (40.8)	1,533 (41.4)	106 (34.4)	
Other	26 (0.7)	22 (0.6)	4 (1.3)	
Missing	1,124 (0)	1,091 (0)	33 (0)	
**Employment**				
Employed	1,091 (45.1)	998 (44.4)	93 (54.4)	.01
Unemployed	1,327 (54.9)	1,249 (55.6)	78 (45.6)	
Missing	2,721 (0)	2,551 (0)	170 (0)	

Abbreviations: CRC, colorectal cancer: IQR, interquartile range (quartile 1–quartile 3).

a Values are no. (%) unless otherwise indicated.

**Table 2:** Clinical Colorectal Cancer Risk Factors of Uninsured Patients Serviced at 9 Free Clinics in Tampa Bay, Florida, 2016–2018^a^

Risk Factor	All Patients (N = 5,139)	No CRC Screening (n = 4,798)	CRC Screening (n = 341)	*P* Value
**BMI, median, kg/m^2^ (IQR)**				
Sample	29.4 (25.4–34.2)	29.3 (25.4–34.2)	29.7 (25.6–34.4)	.94
Missing	1,546 (0)	1,527 (0)	19 (0)	
**Smoking status**				
Active	725 (22.7)	656 (22.6)	69 (23.0)	.09
Past	594 (18.6)	525 (18.1)	69 (23.0)	
Never	1,879 (58.8)	1,717 (59.2)	162 (54.0)	
Missing	1,941 (0)	1,900 (0)	41 (0)	
**Alcohol consumption**				
Active	771 (29.2)	663 (28.0)	108 (39.9)	<.001
Past	192 (7.3)	163 (6.9)	29 (10.7)	
Never	1,676 (63.5)	1,542 (65.1)	134 (49.4)	
Missing	2,500 (0)	2,430 (0)	70 (0)	
**Chronic illness**				
Diabetes	1,455 (28.3)	1,342 (28.0)	113 (33.1)	.047
Inflammatory bowel disease	23 (0.4)	17 (0.4)	6 (1.8)	<.001

Abbreviations: CRC, colorectal cancer; BMI, body mass index; IQR, interquartile range.

a Values are no. (%) unless otherwise indicated.

Of all patients, 341 (6.6%) had a documented CRC screening. Patients with a documented CRC screening were more likely to be employed that those without a screening (54.4% vs 44.4%, *P* = .01) (Table 1). Patients who had a CRC screening were more likely than those without screening to be active (39.9% vs 28.0%) or past (10.7% vs 6.9%, *P* < .001) consumers of alcohol (Table 2). Diabetes was more prevalent among patients who received CRC screening than those without (33.1% vs 28.0%, *P* = .047). IBD was more prevalent among patients with a documented CRC screening than among those without (1.8% vs 0.4%, *P* < .001).

## Discussion

We found a high prevalence of CRC risk factors among uninsured patients in Tampa Bay’s free clinics. More than half of the patients were unemployed and consisted of a largely Hispanic population. We also found a 28.3% prevalence of diabetes and a median BMI of 29.4, suggesting the continued need for management of chronic health conditions.

The prevalence of several known modifiable risk factors for CRC, including smoking, alcohol usage, poor diet, obesity, and lack of physical activity, is higher in low socioeconomic populations (12–14). Hereditary and personal factors associated with CRC include type 2 diabetes, chronic IBD, and family history of CRC (15–18), and many of these risk factors are seen in higher rates within racial and ethnic minority groups (19). A meta-analysis of 29 articles by Luo and colleagues found that type 2 diabetes was associated with a relative risk of 1.37 (95% CI, 1.28–1.42) of developing CRC (15). Notably, previous epidemiologic studies show that Hispanics have a high prevalence of overweight and type 2 diabetes (20,21). 

Patients who are at high risk for CRC and meet US Preventive Services Task Force guidelines are recommended to have routine CRC screening. However, CRC screening compliance remains a challenge in uninsured patients. Shapiro et al reported that 40% of Americans aged 50–75 years had not received recommended CRC screening and that the percentage was higher among those without insurance (80%) (22). Another study by Mojica et al reported that cancer screening rates for Latina women are lower than for non-Latino White women (23). CRC screening rates have been historically lower among Hispanic individuals compared with those who are non-Hispanic White (24). Our results are consistent with the literature, as our patient population was predominantly Hispanic and CRC screening was low, with only 6.6% of patients undergoing routine screening. Additionally, we found that unemployed patients were more likely not to have CRC screening, emphasizing the need for additional resources or better screening strategies for this population. We have previously reported that epidemiologic estimates may be affected by barriers to health care access, such as transportation, work leave, and the severity of disease (25). Furthermore, although colonoscopy is the gold standard for CRC screening, it is expensive. Socioeconomic status may affect providers’ prescription patterns as well as patient compliance. Cheaper alternatives such as the fecal immunochemical test (FIT) and fecal occult blood test (FOBT) can be offered, but these sometimes result in false positive test results (26).

CHCs and FMCs are uniquely positioned to reduce CRC burden because of the large proportion of underinsured and uninsured individuals they serve. Studies show that having a routine source of care is a predictor of CRC test use in these populations (27,28), and several community interventions to increase CRC screening in uninsured patient populations have been successful (29–32). A study by Lairson et al used community health workers, video interventions, or both to increase awareness for colon cancer screening in low-income, uninsured Hispanic patients in El Paso, Texas. These interventions achieved screening rates between 75% and 87% compared with 10% in the comparison group (30). A program for uninsured patients in South Carolina found that FIT screening was more fiscally appropriate for a state’s budget and also an effective choice compared with colonoscopy (29). Patients may experience barriers to access to care, such as lack of transportation, so mail-in FOBT can be used to promote screening in low-income populations (31). One of our contributing clinics in Florida has documented screening rates as high as 64% with the help of a dedicated gastroenterologist and a partnership with the Colon Cancer Alliance (32).

Our study has several limitations, including its retrospective nature and potential for selection bias. Other barriers to health care utilization may exist and may be differentially distributed in the uninsured population, so our study sample may not be representative of the uninsured population at large. Because the clinics operate independently and have different patient health recording methods, we could not collect and analyze certain data, such as diet and exercise. Study variables often contained large numbers of missing values, which could introduce bias beyond that which occurs with the collection of administrative data. Patients who received CRC screening had lower proportions of missing data on risk factors. Patient or provider knowledge of these risk factors may have increased the likelihood of CRC screening (ie, the presence of multiple risk factors was an apparent requisite for screening), and patients with CRC screening may have been more engaged with their primary care center in general. Another consideration is that although our original study focused on a spectrum of chronic diseases, our database did not capture the entire granularity of screening methods (eg, colonoscopy, FIT, FOBT) for CRC.

Nevertheless, our study is among the few that have reported the burden of risk factors and screening rates for CRC in FMCs in Florida (32). Overall, our study further elucidates the disparity of risk factors and CRC burden in the low-income and uninsured population. Because CHCs and FMCs are uniquely positioned to treat and care for this population, our findings should encourage the development of sustainable and cost-effective primary and secondary prevention strategies for this vulnerable group.

### Conclusion

Low-income and uninsured patients of the free clinics in Tampa Bay are at a higher risk of developing CRC because of higher rates of predisposing comorbidities. Continued management of risk factors and increased screening efforts should be made for this vulnerable population. Subsidized screening, including FIT tests and colonoscopies, would strongly benefit these high-risk patients and increase the resources available at free clinics for such preventive measures.
